# Extending *Populus* seed longevity by controlling seed moisture content and temperature

**DOI:** 10.1371/journal.pone.0203080

**Published:** 2018-08-24

**Authors:** Du Hyun Kim

**Affiliations:** Department of Life Resources Industry, Dong-A University, Busan, Republic of Korea; College of Agricultural Sciences, UNITED STATES

## Abstract

The poplar species *Populus davidiana* and *P*. *koreana* are widely grown in plantations and as biofuel resources, but little is known about *ex-situ* seed conservation in poplar. To identify the optimal long-term seed storage conditions for these species, we evaluated the viability of seeds with different seed water contents (SWCs) at various storage temperatures and time periods. *P*. *davidiana* seeds with <6% SWC could be stored at room temperature (RT) for 4 weeks, while *P*. *koreana* seeds showed no storability at RT. *P*. *davidiana* seeds with 3% SWC showed 74% viability after 36 months of storage at 4°C, while those with 9–18% SWC showed >89% viability after 48 months of storage at -18°C. Long-term storage at -80°C was best for *P*. *davidiana* seeds with a wide range of SWCs (3–24%), with 91–98% of normal germination after 48 months of storage. However, the normal germination of *P*. *koreana* seeds with 3–24% SWC declined to <20% after 36 months of storage, even at -18°C and -80°C. No significant difference was observed between seeds immersed vs. not immersed in liquid nitrogen for both species. Our findings increase the possibility for long-term seed conservation for both *Populus* species.

## Introduction

Developing an effective conservation methodology for *Populus* would greatly facilitate basic research and breeding programs and would provide a cost-effective method for seed conservation for long-term storage [1]. The most efficient method for protecting the genetic diversity of plant materials is seed storage [2]. Therefore, more than 1,750 seed banks have been established for *ex-situ* conservation throughout the world [3].

*Populus* seeds maintain viability for only 2 weeks to 1 month under natural conditions, which vary with species, season, and microenvironment. When stored at subfreezing temperatures in sealed containers after proper drying, the viability of *P*. *deltoides* [4, 5], *P*. *tremuloides* [6], and *P*. *grandidentata* [5] seeds has been maintained at fairly high levels for 10 to 12 years at -18°C, with the seed water content (SWC) ranging from 6% to 15%. The loss in viability during these relatively long storage periods varies among and within species [5, 7, 8, 9, 10, 11, 12, 13].

Seed moisture is the most important factor in maintaining seed viability during storage, as it is the primary factor controlling all cellular activities. The metabolic rates of both orthodox and recalcitrant seeds can be minimized by keeping the seeds in a dry state at low temperatures. True orthodox seeds maintained at moisture levels of 5% to 10% can be safely stored at nearly any temperature compared with sub-orthodox or recalcitrant seeds. Therefore, the SWC at the time of storage determines how low the temperature can be set for seed storage; orthodox seeds can be subjected to sub-zero temperatures, whereas recalcitrant seeds cannot. It is not known if sub-orthodox seeds have the same tolerance to low temperatures as orthodox seeds, but they can be stored for a few years at temperatures as low as -20°C [14].

According to previous studies on seed storage behavior in various *Salix* and *Populus* species, the seeds of both genera exhibit orthodox behavior; sub-zero temperatures (-5°C to -20°C) for seeds with 6–10% SWC are usually recommended for long-term storage [15]. *P*. *deltoids* [4, 5], *P*. *grandidentata* [5], and the hybrid poplar *P*. *alba* × *glandulosa* [11] show orthodox behavior, whereas *P*. *nigra* seeds are sub-orthodox. By contrast, for recalcitrant species such as *P*. *laurifolia*, *P*. *canescens*, and *P*. *tremula*, the maximum advisable storage period is 4 weeks under non-drying conditions at 0°C [16]. For *S*. *caprea*, seeds with identical collection dates and sites from the same clone can show different storage behaviors. Therefore, paternal × environmental factors influence seed behavior during desiccation and storage [10].

Cryopreservation is currently the only effective option for long-term conservation of intermediate and short-lived seeds [17, 18, 19, 20]. Even though cryostorage maintains the viability of *Salix* and *Populus* seeds longer than other temperature conditions, aging is not completely stopped, and seed longevity is shorter than that predicted for many other species. For example, germination rates of only 19% and 33% were observed in seeds after 20 years of cryostorage (initial germination rates were 90% and 100%). Variation in seed storability under cryopreservation has been detected among *Salix* species of different accessions with different levels of initial seed quality [13].

In recent years, *Populus* are to a certain extent in commercial use in Europe, North America, and Asia in short rotation forestry management regime by the growing demand for biomass for energy. In Hungary, poplar forest accounts for 10% (1.9 million ha) of the total forest area. Sweden has increased the interest in poplars species suitable for short rotations with about 1744 ha of *Populus* plantations in 2014. The United States is conducting poplar research to replace 30% of its gasoline consumption with woody biofuels and to expand carbon sinks [21, 22]. The genus *Populus* has been reported as economically important for wood productions for industrial purposes such as plywood, sawn timber and pulpwood, fuelwood and biomass for energy. It also roles environmentally important plays for the erosion control, wild habitat, restoration of natural river-bank environments, soil remediation, and carbon sequestration for the reduction of the effects of climate change and air pollution [23–25]. The genus *Populus* has been reported as economically important for wood productions for industrial purposes such as plywood, sawn timber and pulpwood, fuelwood and biomass for energy. It also roles environmentally important plays for the erosion control, wild habitat, restoration of natural river-bank environments, soil remediation, and carbon sequestration for the reduction of the effects of climate change and air pollution [23–25]. There are approximately 30 *Populus* species in the temperate zone of the Northern hemisphere. Five of these species, *P*. *davidiana*, *P*. *maximowiczii*, *P*. *koreana*, *P*. *simonii*, and *P*. *tomentiglandulosa*, are found in the Korean peninsula. *P*. *davidiana* is a deciduous broadleaved tree species originating in Korea that grows well in slightly dry, acidic, harsh mountainous areas over 500 m above sea level. *P*. *davidiana* is an excellent species for mountain planting. Studies have been carried out to select high-quality, superior trees that are highly adaptable to mountain plantations, considering propagation methods as well as disease tolerance [26, 27]. *P*. *koreana*, which also originated in Korea, grows in areas 400–1600 m above sea level. In South Korea, a wild population of *P*. *koreana*, which was thought to have almost disappeared, was discovered in the valley of Odae Mountain in Gangwon Province in 2012. *P*. *koreana* is valuable because it grows rapidly (at a rate of 2–3 m per year) and is amenable to mass propagation. *P*. *koreana* trees are 25 m tall with a diameter of 1 m and a dioecious reproductive system. These trees flower in May and produce ripe capsules in June. *P*. *koreana* is highly valued not only as an environmentally friendly source of biofuels and as a carbon sink, but also for pulp and paper production [28]. These two poplar species are of interest in Korea for their possible use in the production of bioenergy sources such as bio-ethanol, and studies are underway to improve their breeding. However, the natural ranges of these species are shrinking as a result of climate change, and there is an urgent need to develop effective preservation technologies to protect these natural resources. Limited information is available about the storage of *Populus* species, and the use of mechanical freezing has only been examined for short storage periods or for a limited range of *Populus* species. Additionally, the optimal storage conditions for *P*. *davidiana* and *P*. *koreana* have not been determined.

No single method of storage is generally acceptable for all *Populus* species [29], and therefore studies are needed to develop storage protocols for these species, such as determining the optimal SWC and temperature conditions for specific species. The objective of this study was to examine the differences in seed viability associated with different storage conditions for *P*. *davidiana* and *P*. *koreana*. Specifically, we investigated (1) whether *Populus* seed viability could be retained after a storage period of 48 months in a mechanical freezer, (2) how storage temperature and SWC affect seed storability, and (3) whether optimal SWC ranges could be identified for LN (liquid nitrogen) storage. The results of this study are important for developing protocols for medium- and long-term storage of *P*. *davidiana* and *P*. *koreana* seeds using mechanical freezing and cryopreservation methods.

## Materials and methods

### Plant materials, catkin collection, and seed cleaning

Catkins were collected when cotton emerged from partially opened capsules. Catkins from *Populus davidiana* Dode were collected in an artificial plantation (37°15´N 126°57´E, 40.5 m elevation, Kyunggi Province, Republic of Korea) on May 16, 2013. Catkins from *Populus koreana* Rehder were collected in a natural forest in Mt. Odae (37°44´N 128°35´E, 705 m elevation, Gangwon Province, Republic of Korea) on June 11, 2013. Catkins were collected from several trees growing within a 50 m area with no other *Populus* species located nearby to limit the potential for cross-pollination. Samples were transported to the laboratory and processed immediately. Seed collection was conducted by National Institute of Forest Science that had responsible for the management of artificial plantation (*P*. *davidiana*) and got field study permission (*P*. *koreana*) from Korea National Park. This field study did not involve endangered or protected species. To open the capsules fully, the catkins were arranged in a single layer and dried for 2 days at ambient temperature or in an incubator for 2 days. Seeds were cleaned from the cotton using air and soil screens as described by Dreesen [30]. The distribution, ecological characteristics, and seed characteristics of the two species are described in Table 1.

**Table 1:** Species characteristics of *P*. *davidiana* and *P*. *koreana*.

Species characteristics	*P*. *davidiana* Dode	*P*. *koreana* Rehder
Native occurrence	Korea, Far east Asia, China, Japan	Korea, China, Russia
Distribution in Korea	Hamkyungdo, Pyeongando, GangWondo province, Northern Gyeonggido	Northern part of South Korea, North Korea
Altitude	100–1900 m	400–1600 mm
Catkins	2–6 mm, long oval	Wide ovate
Seed ripening and dispersal	May	June
Cleaned seeds dry weight (g)	3335±84 seeds/g DW	4166±165 seeds/g DW
Seed size (length × width, mm)	1.44 (±0.12) × 0.73 (±0.07)	1.69(±0.12) × 0.64 (±0.07)

### Seed desiccation and storage conditions

Catkins were placed in an open dish and dried in an incubator at 20°C in the light for 2 days to make the catkins open. *P*. *davidiana* seeds (3335±84 seeds·g^-1^ dry weight) were brown and smaller but heavier than *P*. *koreana* seeds (4166±165 seeds·g^-1^ dry weight), which were greenish, larger, and lightweight. The seed coats of *P*. *davidiana* were firm, whereas those of *P*. *koreana* were soft and shrunk after desiccation (Table 1). After the catkins opened at 20°C and the seeds were cleaned from the cotton, fresh seeds had a SWC of 9.6% and 9.9% for *P*. *davidiana* and *P*. *koreana*, respectively. After the seeds were separated from the cotton, they were stored above 100 g of fresh silica gel for desiccation or distilled water in sealed plastic containers (L110 × W110 × H35 mm) for rehydration at 20°C. SWC was reduced to 3–24% after 2–4 days of incubation with silica gel or rehydration. Seeds that had been dried or rehydrated to 3%, 6%, 9%, 12%, 18%, 21%, and 24% SWC were sealed in two aluminum bags within a polythene bag and stored at several different temperatures: RT (~25°C), 4°C, -18°C, -80°C, and -196°C (LN). Further details about the desiccation and rehydration procedures are described in Popova et al. [10, 11]. Water content was monitored during desiccation and hydration treatments and determined gravimetrically (International Seed Testing Association 2009) for samples weighting 20 mg each after drying at 130°C for 1 h. Water content was evaluated as the average of three replications [10, 11, 31]

*P*. *davidiana* and *P*. *koreana* seeds were used to evaluate seed longevity at RT (~25°C). Seeds were stored at RT for 4 and 3 weeks for *P*. *davidiana* and *P*. *koreana*, respectively.

The effects of mechanical cooling at low temperature (4°C, -18°C, and -80°C) were investigated using seeds from both *Populus* species. Germination was evaluated after storage periods of 5, 12, 24, 36, and 48 months for *P*. *davidiana* and 12, 24, 36, and 48 months for *P*. *koreana*.

For cryopreservation, seeds were placed into 2 ml plastic cryo-ampoules (Nalgene, USA), which were sealed and plunged in LN. After 1 week of storage at -196°C, the ampoules were rewarmed in a water bath at 37°C for 90 s.

### Seed germination test

Seeds were placed on top of two layers of filter paper moistened with distilled water in 90 mm Petri dishes at 20±1°C for 24 h under constant light. Germination was checked daily for 7 days and assessed when normal and abnormal seedlings could easily be distinguished. Seed viability can calculated in terms of survival (total germination: radicle penetration from seed coat) and normal seedling growth (normal germination). Normal germination that produced seedlings with cotyledons, a hypocotyl, and roots were considered as germination in this study, as described for *Salix* and *Populus* species [10, 11, 13, 32]. For each combination of seed moisture content, temperature, and storage period, germination was assessed in four replicates containing 25 seeds.

### Statistical analysis

Statistical analyses were conducted using SAS software (SAS Institute, USA). Generalized linear models was used to assess the influence of storage temperature, SWC, and storage period on normal germination. Means were compared using the Duncan multiple range test (DMRT) at the 5% level. Significant differences between non-treated (-LN) and cryopreserved (+LN) seeds were compared using a Student’s t-test.

## Results

### Germination of *Populus* seeds during storage at room temperature

**Fig 1:**
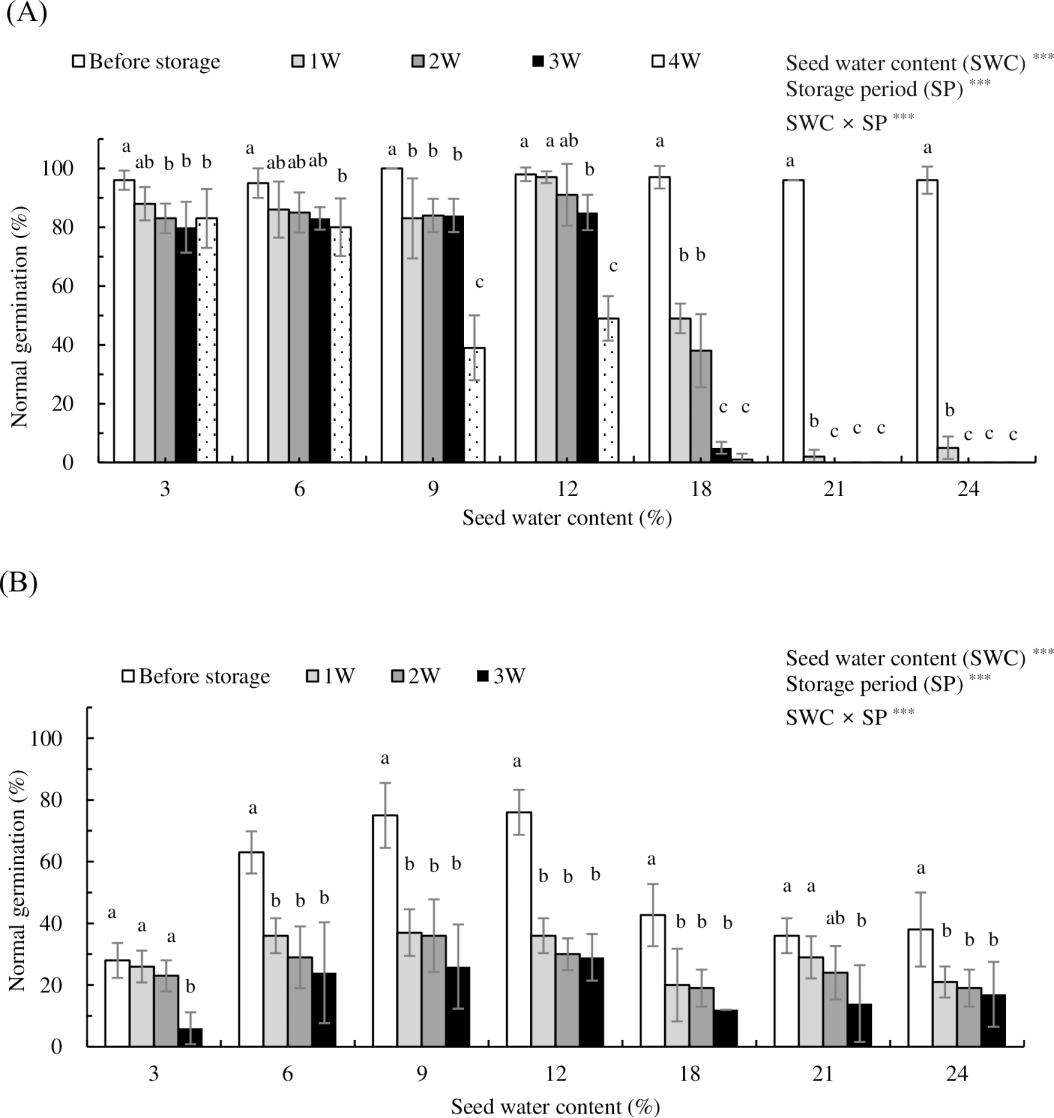
**Rates of normal seed germination before storage (0 W) and after 1–4 weeks (1 W–4 W) of storage at room temperature for *P*. *davidiana* (A) and *P*. *koreana* (B). Seed water contents were 3%, 6%, 9%, 12%, 18%, 21%, and 24% (fresh weight basis).** Different letters indicate statistically significant differences among five storage periods within each seed water content at p<0.05, according to Duncan’s multiple range test. Vertical bars represent means of four replicates ± SD (n = 4). ***p<0.001.

Catkins were placed at ambient temperatures for 2 days to allow for catkin opening and air separation of seeds. Under these conditions, 97% and 87% normal germination were observed in *P*. *davidiana* and *P*. *koreana*, respectively (Table 2). Adjusting SWC influenced seed viability in both *Populus* species (Fig 1 and Table 2). Seed storability at room temperature (RT) for *P*. *davidiana* and *P*. *koreana* was examined. Seeds from these species exhibited different levels of storage stability at RT with respect to SWC. *P*. *davidiana* seeds exhibited normal germination ranging from 95% to 100% in seeds at all SWCs before storage (Fig 1A and Table 2). However, seed viability was significantly reduced after 1 week of RT storage compared with germination before storage, depending on SWC (p<0.05). Seeds with 3%, 6%, and 9% SWC exhibited a slight decline in germination (to 88%, 86%, and 83%, respectively) after 1 week of storage at RT. However, seeds with 18%, 21%, and 24% SWC exhibited a sharp decrease in germination (to 49%, 2%, and 5%, respectively) after 1 week of storage at RT. No significant changes in germination levels after 1 week of storage were observed for seeds with 12% SWC (97%). After 2 weeks of storage, a dramatic reduction in germination was observed at >18% SWC. Seeds with 18%, 21%, and 24% SWC exhibited normal germination of only 38%, 0%, and 0%, respectively, after 2 weeks of storage at RT. At 3 weeks of storage at RT, seeds with 3–12% SWC showed 80–85% normal germination, whereas seeds with 18% SWC germinated at a rate of less than 5%, and seeds with 21% and 24% SWC failed to germinate. After 4 weeks of storage at RT, seeds with 3% and 6% SWC germinated at a rate of 83% and 80%, respectively, and seeds with 9% and 12% SWC germinated at a rate of 39% and 49%, respectively, whereas seeds with 18%, 21%, and 24% SWC germinated at a rate of only 0–1% (Fig 1A).

**Table 2:** Effects of seed water content (SWC) and cryopreservation (LN) on normal seed germination in *Populus davidiana* and *P*. *koreana*.

Seed water content (SWC, %)	Normal germination (%)			
	*P*. *davidiana*		*P*. *koreana*	
	-LN	+LN	-LN	+LN
After seed cleaning	97 ± 3.3		87 ± 9.7	
3	96 ± 3.3^ns z^	84 ± 7.3^c^	28 ± 5.7^c^	27 ± 7.6^c^
6	95 ± 5.0	100 ± 0.0^a^	63 ± 6.8^a^	67 ± 9.2^a^
9	100 ± 0.0	95 ± 3.8^ab^	75 ± 10.5^a^	78 ± 12.4^a^
12	98 ± 2.3	98 ± 2.3^ab^	76 ± 7.3^a^	72 ± 6.5^a^
18	97 ± 3.8	93 ± 6.8^ab^	43 ± 10.1^b^	43 ± 11.0^b^
21	96 ± 0.0	90 ± 5.2^bc^	36 ± 5.7^bc^	38 ± 2.3^bc^
24	96 ± 4.6	90 ± 5.2^bc^	38 ± 12.0^bc^	38 ± 5.2^bc^
Significance				
SWC	***		***	
LN	**		ns	
SWC × LN	*		ns	

Values are means of four replicates ± SD (n = 4). Different letters indicate statistically significant differences among seed water content within non-treated (-LN) or cryopreserved (+LN) seeds at p<0.05, according to Duncan’s multiple range test in each species. ^z^No significant different between non-treated (-LN) and cryopreserved (+LN) seeds in both species.

*, **, and *** indicate significant difference at p<0.05, p<0.01, and p<0.001, respectively, and ns indicates non-significance.

*P*. *koreana* seeds yielded 87% normal germination prior to seed moisture adjustment by desiccation or rehydration (Table 2). After SWC was adjusted to a range of 3% to 24%, the normal germination decreased to 28% to 76%. The normal germination of *P*. *koreana* decreased significantly with increasing storage time at RT for seeds at all seven levels of SWC (p<0.05). Seeds with 6–12% SWC exhibited the slowest deterioration over time, with a normal germination decreasing to 36–37% after 1 week of storage but slowly decreasing to 24–29% after 3 weeks of storage at RT (Fig 1B).

These data suggest that *P*. *davidiana* seeds survive longer than *P*. *koreana* seeds at RT. Moreover, analysis of variance showed that seed water content (SWC), storage period (SP), and SWC × SP interactions were significant for normal germination in both species. *P*. *davidiana* seeds with <6% SWC could be stored for 4 weeks at RT, while *P*. *koreana* seeds with 6–12% SWC could be stored 1 weeks at RT (Fig 1 and Table 3).

**Table 3:** Summary of optimal seed storage conditions for each species of *P*. *davidiana* and *P*. *koreana*.

Storage temperature (°C)	Species			
	*P*. *davidiana*		*P*. *koreana*	
	Optimal SWC range (%)	Storage period (months); NGP	Optimal SWC range (%)	Storage period (months); NGP
Before storage (NGP)	97%		87%	
Room Temperature	< 6	1; 80–83%	6–12	0.25; 36–37%
4	3	5; 93–100%	12	12; 27%
-18	12	48; >89%	9–12	24; 26–28%
-80	3–24	48; >91%	9–12	24; 28–29%
-196	6–18	NA; 93–100%	6–12	NA; 67–78%

Normal germination percentage (NGP); Seed water content (SWC); Not available (NA)

### Effects of SWC, storage temperature, and period on seed viability

**Fig 2:**
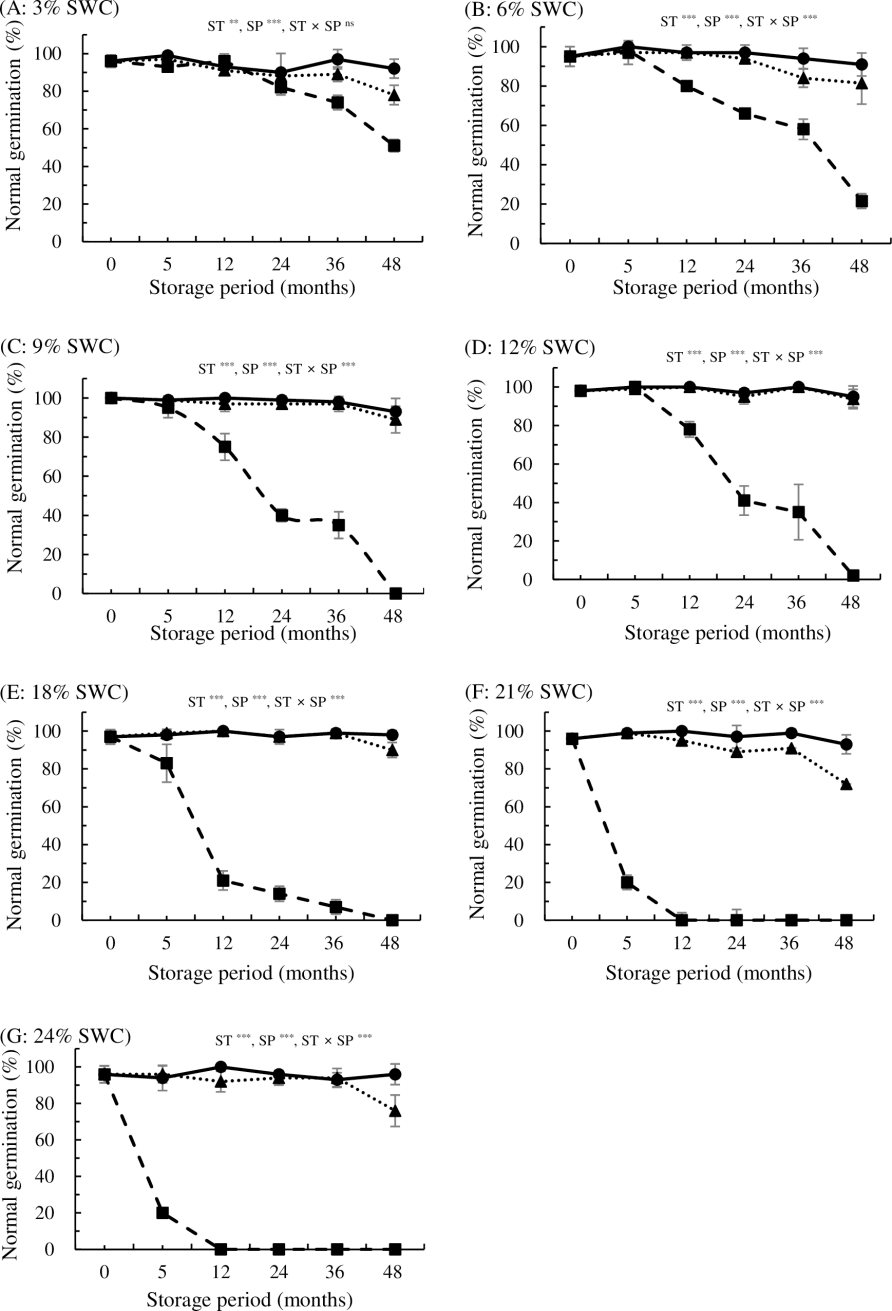
**Effects of storage temperature at 4°C (■), -18°C (▲), and -80°C (●) on normal germination for *P*. *davidiana* seeds stored for up to 48 months with water contents adjusted to 3% (A), 6% (B), 9% (C), 12% (D), 18% (E), 21% (F), or 24% (G) (fresh weight basis).** Storage temperature (ST) ***, seed water content (SWC) ***, storage period (SP) ***, ST × SWC ***, ST × SP ***, SWC × SP ***, ST × SWC × SP ***. All values are means of four replicates ± SD (n = 4). *** indicate significant difference at p<0.001.

The effects of SWC on seed longevity after storage at 4°C, -18°C, and -80°C was investigated. Significant effects of SWC, storage temperature, storage period, and their interactions were detected for *P*. *davidiana*, as shown in Fig 2 (p<0.05). Longevity varied among seeds with different SWCs after storage at 4°C. No significant changes in normal germination were observed for desiccated seeds ranging from 3% to 12% SWC stored at 4°C for 5 months (93–100%). The normal germination declined to 83%, 20%, and 20% after 5 months of storage at 4°C for seeds with 18%, 21%, and 24% SWC, respectively. Seeds with 3% and 6% SWC germinated at a rate of up to 74% and 58%, respectively, after 36 months of storage at 4°C. By contrast, normal germination rates of seeds with 9%, 12%, 18%, 21%, and 24% SWC sharply decreased to 35%, 35%, 7%, 0%, and 0%, respectively, after 36 months of storage. Longer periods of storage (48 months), however, significantly decreased the germination rates of seeds with 3% and 6% SWC to 51% and 22%, respectively. The normal germination of other seeds with >9% SWC dropped to 0% after 48 months of storage at 4°C. On the other hand, a lower storage temperature extended seed longevity. Seeds with 9%, 12%, and 18% SWC exhibited >89% germination after 48 months of storage at -18°C, whereas seeds with all seven levels of SWC germinated at a rate of >91% after 48 months of storage at -80°C. Therefore, a storage temperature of -80°C is best for long-term storage of *P*. *davidiana* over a wide range of SWCs (3–24%; Fig 2).

**Fig 3:**
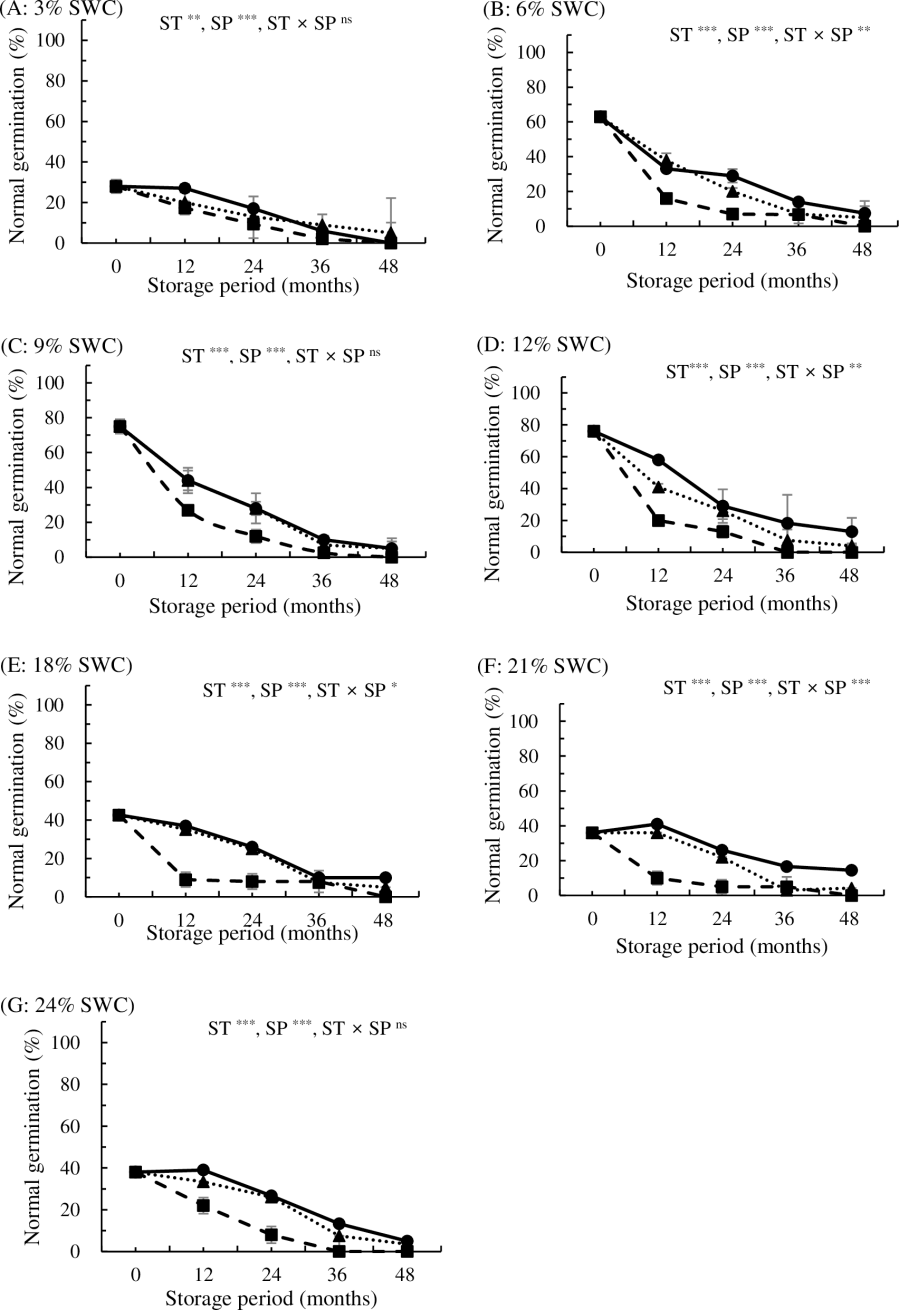
**Effects of storage temperature at 4°C (■), -18°C (▲), and -80°C (●) on normal germination in *P*. *koreana* seeds stored for up to 48 months with water contents adjusted to 3% (A), 6% (B), 9% (C), 12% (D), 18% (E), 21% (F), or 24% (G) (fresh weight basis).** Storage temperature (ST) ***, seed water content (SWC) ***, storage period (SP) ***, ST × SWC **, ST × SP ***, SWC × SP ***, ST × SWC × SP ^ns^. All values are means of four replicates ± SD (n = 4). ** and *** indicate significant difference at p<0.01 and p<0.001, respectively, and ^ns^ indicates non-significance.

Seed germination in *P*. *koreana* was also significantly affected by SWC, storage temperature, and storage period (p<0.05) (Fig 3). However, significant interactions between these three factors were not observed via GLM. Normal germination of *P*. *koreana* seeds with increasing storage temperature and period. At all seven levels of SWC, the normal germination of seeds stored at 4°C decreased to 9–27% after 12 months of storage and to 5–13%, 0–8%, and 0% after 24, 36, and 48 months of storage, respectively. Seed with 3–24% SWC stored at -18°C exhibited slightly higher normal germination of 13–32% compared with those stored at 4°C for 24 months; however, their germination dropped to <10% after 36 months of storage. Seed with 3–24% SWC stored at -80°C exhibited normal germination of 27–58%, 17–29%, 6–18%, and 0–14% after 12, 24, 36, and 48 months of storage, respectively. In conclusion, *P*. *koreana* seeds had very poor storability, even after adjusting SWC and storage at low temperature (Fig 3).

Based on the above results, the storage life of poplar seeds significantly differs depending on species. The longevity of *P*. *davidiana* seeds was extended under low temperatures and optimum moisture contents. The optimum moisture content of seeds varied with storage temperature, i.e., 3% SWC at 4°C and 12% SWC at -18°C. No loss of viability was observed for *P*. *davidiana* seeds stored at -80°C for up to 48 months for all SWCs. Seed viability in *P*. *koreana* decreased to 26–29% after 24 months of storage, even at sub-zero temperatures in seeds with 9% and 12% SWC (-18°C and -80°C). Even though we identified a wider safe range of SWCs (6–24%) in *P*. *koreana* stored at lower temperatures, the germination rate dropped to <20% after 36 months of storage at -80°C for all SWCs (Table 3).

### Effects of SWC and cryopreservation on two *Populus* species

The responses of freshly collected *P*. *davidiana* and *P*. *koreana* seeds to desiccation and subsequent cryopreservation were tested. Storing *Populus* seeds cryogenically in LN was found to be effective and feasible. SWC had significant effects on seed germination in two species. However, LN and SWC × LN interaction had significant effect on normal seed germination in *P*. *davidiana*, but not in *P*. *koreana*. (Table 2).

After LN storage, seeds with 3%, 6%, 9%, 12%, 18%, 21%, and 24% SWC germinated at a rate of 84%, 100%, 95%, 98%, 93%, 90%, and 90%, respectively, in *P*. *davidiana*. No significant difference was observed between seeds not immersed in LN (-LN) and seeds immersed in LN (+LN) in *P*. *davidiana*, as determined by t-test. The normal germination of *P*. *koreana* seeds varied depending on SWC; however, no significant difference was observed among +LN seeds with different SWCs. Additionally, normal germination showed no significant difference between–LN and +LN within the same SWC as determined by t-test (p<0.05).

For both species, SWC had a significant effect on germination. However, the normal germination was unaffected by SWC when seeds were cryopreserved within a range of 3–24% SWC, as determined by comparing germination in -LN vs. cryopreserved seeds (p<0.05). Cryostorage had no effect on germination in *P*. *davidiana* and *P*. *koreana* in seeds with 3–24% SWC (Table 2). Considering of normal germination production, 6–18% and 6–12% of SWC recommended for LN storage in *P*. *davidiana* and *P*. *koreana* (Table 3). These results emphasize the importance of further characterizing the effect of SWC on seed viability and elucidating safe SWC ranges for cryopreservation for individual *Populus* species.

## Discussion

### Effect of SWC on seed viability in seeds stored at RT

In this study, it was found that the seed storability of *P*. *davidiana* at RT varied with respect to SWC (Fig 1). Seeds with 3% and 6% SWC exhibited 83% and 80% normal germination after 4 weeks of storage at RT, respectively (97% normal germination prior to storage). On the other hand, seeds with 21% and 24% SWC exhibited rapid and significant reductions in normal germination to only 2% and 5% after 1 week of storage at RT. Indeed, ripe fertile *P*. *grandidentata* seeds have an extremely high germination capacity (99%), but the seeds are viable under natural conditions for no more than 2 or 3 weeks [33]. *P*. *alba* seeds, which have high germinability, germinated at a rate of 92% immediately after collection, and most (80%) remained viable for 2 weeks after they were released from the catkins. The median seed longevity (G_50_) of *P*. *alba* is 30 days, indicating potential for survival under dry conditions well beyond 1 month. Some seeds still germinated after 70 days (10 weeks) of storage after the capsules had opened [34]. In the current study, the germination of both *P*. *davidiana* and *P*. *koreana* seeds was affected by moisture content and storage period at RT. *P*. *davidiana* seeds with <6% SWC could be stored for 4 weeks at RT, whereas *P*. *koreana* seeds showed no storability at RT. Similar to observations for *P*. *koreana*, *P*. *deltoids* seeds remain viable for only 1–2 weeks [35, 36, 37]. Suszka et al. [12] reported that seeds (9% SWC) that were stored at 20°C exhibited a significant and dramatic decrease in total germination. After 1 month, the germination dropped from 91% to 22%, and after 3 months, only 1% of *P*. *nigra* seeds germinated.

During seed storage, seeds deteriorate, lose vigor, and, as a result, become more sensitive to stress during germination and ultimately die. The rate of this aging process depends on the SWC, temperature, and initial seed quality [38, 39]. Seed longevity is a quantitative trait for which variation is present among naturally occurring accessions in Arabidopsis, lettuce, and rice [40]. Vast variations in seed weight, size, and germination have also been reported among clones in tree species such as *Dalbergia sissoo* [41], *Juniperus procera* [42], and *Santalum album* [43]. In addition, Popova et al. [10] reported variation in seed longevity among the same clones in *S*. *caprea*. Therefore, both genetic and environmental factors for each species should be considered when investigating seed longevity.

In this study, the high-viability *P*. *davidiana* seeds showed extended viability with decreasing SWC after 4 weeks of storage at RT. The low-viability *P*. *koreana* seeds produced normal seedlings at relatively high rates from seeds with lower SWC (6–12% SWC) compared with seeds with 3% and 18–24% SWC stored at RT. There are many reports of the rapid loss of viability in *Populus* seeds stored at RT, but the varying effects of a wide range of SWCs were not observed in seeds under RT storage. Based on the *Populus* species examined in this study, it appears that the optimum SWC for storage of low-viability seeds at RT is high compared with the optimum SWC for highly viable *Salix* seeds stored at RT reported by Popova et al. [10, 11]. Results from this study are similar to those of Maroder et al. [32], who found that germination of a high-vigor lot of *Salix* seeds (100% initial normal germination) was not affected by dehydration to 6.7% SWC, but germination decreased with further dehydration to 4.3% SWC. The lower vigor lot (75% initial normal germination) was more susceptible to dehydration, and germination decreased following dehydration to 6.7% SWC in *S*. *alba*. Additionally, based on this study findings that SWC >12% accelerated the reduction in seed viability at RT, a low SWC of 6% is recommended for efficient long-term storage of *P*. *davidiana* and *P*. *koreana* seeds at RT.

### Responses of seeds to storage temperature, storage period, and SWC

Most previous reports on seed longevity in *Populus* recommend sub-zero temperatures with 6–13% SWC for long-term storage [11, 14, 15]. In the current study, significant effects of SWC, storage temperature, storage period, and their interactions in two *Populus* species were observed.

No significant changes in germination level were observed for seeds with 3% SWC stored at 4°C for 12 months (96% germination). However, the normal germination decreased with increasing storage period. Similarly, desiccated *P*. *nigra* seeds (0.07 g g^-1^ SWC, 7.1%) remain viable at 3°C for 12 months [14].

Similar to the results for seeds stored at RT, seeds with lower SWC (3–6%) retained higher viability (51% and 22%) than those with higher SWC (9–24%), which failed to germinate (0% germination) after 48 months of storage at 4°C. Suszka et al. [14] also reported that seeds stored at 3°C deteriorated more rapidly at 0.15 g g^-1^ (13%) SWC than at 0.07 g g^-1^ (7.1%) SWC in *P*. *nigra*. Moreover, lower storage temperatures extended seed longevity in *P*. *davidiana*. Seeds with 9%, 12%, and 18% SWC germinated at a rate >89% at -18°C, whereas seeds with all seven SWC levels had germination rates >91% at -80°C, after 48 months of storage. Storage at lower temperatures results in higher levels of germination in *S*. *alba* and *S*. *matsudana* [32]. In orthodox *P*. *deltoides* seeds, a 21% loss in viability was recorded after 6 years of hermetic storage at -20°C for seeds with 6–10% SWC [4]. *P*. *balsamifera* seeds retained high viability after 3 years of storage at -10°C (no recommended SWC was given) [9]. Similarly, Wang [5] reported a 24–100% loss in viability in *P*. *deltoides* seeds with 8.4–13.5% SWC after 10 years of storage at -18°C. The viability of *P*. *grandidentata* seeds with 10.8–14.8% SWC decreased by 14–29% after 12 years of hermetic storage at -18°C [5].

Seed germination in *P*. *koreana* was also significantly affected by SWC, storage temperature, and storage period (p<0.05) (Fig 3). In contrast to *P*. *davidiana*, the normal germination of *P*. *koreana* seeds stored at 4°C for 12 months dropped to 9–27% for all seven levels of SWC (initial germination rate of 87%). Seeds stored at lower temperatures retained higher viability. Seeds with all SWCs (3–24% SWC) stored at -18°C and -80°C exhibited slightly higher levels of normal germination (20–44% and 27–58%, respectively) after 12 months of storage. The optimal SWCs of *P*. *koreana* for 24 months storage were 9%, 9–12%, and 6–24% for storage temperature at 4°C, -18°C, and -80°C, respectively. Low-temperature storage (-80°C) alleviated the effects of SWC on seed storage, allowing for a wide range of safe SWCs. Low storage temperatures increased storability in both *Populus* species, but the optimal SWCs differed depending on the species.

In the current study, seeds of different species responded differently to desiccation and hydration. This finding suggests that the variability in seeds of different species, SWCs, and seed lot quality precludes the development of a uniform storage method, as mentioned by Popova et al. [11]. The results indicate that other factors, such as seed maturity, may have a significant impact on seed viability and storage behavior. The seed coats of mature seeds are firm, while immature seeds have soft, easily depressed seed coats. Seed maturation in *P*. *ciliata* is indicated by seed color and (more importantly) seed moisture content and the opening of capsules [44]. Similarly, in the current study, *P*. *koreana* seeds had soft seed coats, low weights, and light colors compared with *P*. *davidiana*. Seeds collected early are immature and exhibit poor germination and storability [13, 39].

The *P*. *davidiana* seeds investigated in the current study can be classified as short-lived, since dried seeds lost viability within 5–12 months of storage in a refrigerator. The current study demonstrated that low temperature (such as -80°C) is a more important factor than SWC for the storage of *P*. *davidiana* and *P*. *koreana*, as reported by Suszka et al. [12] for *P*. *nigra* seeds. The current results indicate that *P*. *davidiana* seeds should be classified as orthodox because they can be stored for 4 years after desiccation at a wide range of SWCs (3–24%) at -20°C and -80°C with little or no decrease in germination. However, the germinability of *P*. *koreana* seeds declined after desiccation or rehydration. Additionally, germinability declined at 4°C and began to decline at -20°C after 24 months of storage. Therefore, this study suggest that these seeds could be classified as intermediate rather than orthodox, which is similar to *P*. *nigra* seeds [12]. Nevertheless, the present results show that not only desiccation and temperature tolerance but also the broader aspects of seed maturation need to be considered to assign the proper category. Bonner [14] classified poplar (*Populus* L.) seeds as sub-orthodox, and an earlier study indicated that the viability of seeds can be maintained for several years when they are stored at below-freezing temperatures in a dry atmosphere [45]. No single method of storage is generally acceptable for all *Populus* species [46]. The disagreement about the assignment of *Populus* species to a specific seed category (recalcitrant, intermediate, orthodox) and the lack of comprehensive information about suitable storage protocols make it difficult to design ways to preserve *Populus* seeds. To prolong seed longevity in *P*. *davidiana* and *P*. *koreana*, the storage of mature seeds at low temperatures is mandatory.

### Cryopreservation of *P*. *davidiana* and *P*. *koreana* seeds

The current study demonstrated that low temperature is a more important factor than SWC in the storage of *P*. *davidiana* and *P*. *koreana* seeds. The maximum storage period reported for most *Populus* species does not exceed 8–12 years at -18°C and -20°C. Therefore, cryogenic storage would be a suitable method for long-term conservation, as recommended by Pritchard [20] for short-lived seeds. In this study, storage at lower temperatures resulted in higher levels of germination for seeds at various SWCs in both species, which is in agreement with the results of Maroder et al. [32] for *S*. *alba* and *S*. *matsudana* seeds. Both high- and low-viability lots of *S*. *gracilistyla* and *P*. *alba* × *P*. *glandulosa* seeds show very similar hydration windows for cryopreservation [11]. In the current study, no significant difference was observed between non-LN immersed (-LN) and LN immersed (+LN) seeds for both *P*. *davidiana* and *P*. *koreana*. Seed lots of *S*. *hallaisanensis* and *S*. *gracilistyla* with 10% SWC (with ~80% germination) survived cryopreservation, but those with lower SWC were more sensitive to this treatment. By contrast, *Populus* seeds had greater desiccation tolerance combined with cryopreservation capability than *Salix* seeds. The hydration window of 0.14–0.31 g g^-1^ (~12.3−23.1% SWC) determined for *S*. *hallaisanensis* occurred at the upper limit of the SWC range among the species tested and resembled those obtained for low-viability seeds of a *S*. *caprea* clone at SWCs of 0.2–0.37 g g^-1^ (~16.7−27% SWC) [10]. Popova et al. [11] found that hybrid poplar seeds (*P*. *alba* × *P*. *glandulosa*) survived 2 weeks of storage in LN at SWCs ranging from 0.07 to 0.10 g g^-1^ (~6.5−9.1% SWC) without a significant loss in germinability [10]. Suszka et al. [12] demonstrated that *P*. *nigra* seeds maintained high levels of germination even after 2 years of cryostorage for both fresh (0.15 g g^-1^, 13%, SWC) and desiccated (0.07 g g^-1^, 7.1% SWC) seeds. Maroder et al. [32] reported that 11 months of cryostorage did not adversely affect the germination of *S*. *matsudana* seeds. Pence [17] reported that *P*. *deltoides* seeds can be cryostored when their SWC ranges from 0.09 to 0.17 g g^-1^ (8.1–14.5%). Analysis of water status and the thermal behavior of low-viability *Salicaceae* seeds during cooling and rewarming procedures is a promising approach [47] for investigating the mechanisms underlying the unusually high safe SWCs of some *Salix* and *Populus* species. Ballesteros and Pence [13] reported that aging does not completely stop and seed longevity is shorter than predicted for many other species under cryostorage. *P*. *deltoides* was not affected by drying or LN exposure. While seeds of *P*. *deltoides* accessions stored in LN showed 19% and 33% germination after 20 years of storage (initial normal germination of 90% and 100%, respectively), no seeds stored at 4°C or -20°C during the same time period were viable. A high initial seed quality is important for obtaining the maximum benefits of cryostorage. For *Salix* species, the lowest quality seeds showed the lowest viability after LN storage (0% to 64%), and the highest quality seeds showed the highest viability after LN storage (76% to 96%). However, for *P*. *deltoides* accessions that had been stored for 15 and 20 years in LN, differences in germination after LN storage appear to be related to storage time and not to seed quality. For *Salix* species, differences in storage time between accessions did not explain their differences in viability after LN storage. In the current study, the responses of seeds with 3–24% SWC to 1 week of LN exposure were tested and compared their responses for LN. Therefore, cryostorage could indeed decrease seed viability after 15 or 20 years, as reported by Ballesteros and Pence [13]; however, cryostorage maintained the viability of *Populus* seeds longer than other temperatures in a mechanical freezer.

## Conclusion

The seed storability of two *Populus* species differed based on species, and their optimum storage conditions were different. The optimum storage conditions of the two species showed significant differences according to SWC and storage temperature. *P*. *davidiana* seeds tolerated desiccation to 3% SWC, and seed longevity was greater at lower storage temperatures. According to the criteria for orthodox seeds [48], we conclude that the *P*. *davidiana* seeds investigated showed orthodox seed storage behavior. By contrast, my results suggest that *P*. *koreana* seeds can be classified as sub-orthodox or intermediate. *P*. *davidiana* seeds with <6% SWC could be stored for 4 weeks at RT. A storage temperature of -80°C was best for long-term storage of *P*. *davidiana* at a wide range of SWCs (3–24%). However, even though we identified a wider safe range of SWCs (6–24%) for *P*. *koreana* at lower temperatures, the germination rate dropped to <20% after 36 months of storage for seeds with 3–24% SWC, even at -18°C and -80°C. *P*. *davidiana* seeds at various SWCs could be stored long-term without deterioration in a mechanical freezer; however, this treatment was less suitable for *P*. *koreana* seeds, since they only germinated at a rate of 13% and 14% for seeds with 12% and 21% SWC, respectively, after 4 years of storage at -80°C. Therefore, other methods, such as cryogenic storage, are needed to extend the storage time of *P*. *koreana* seeds. Cryopreservation of *Populus* seeds can be used as a backup method to traditional mechanical freezer storage in seed banks and is the method of choice for long-term conservation for up to 15 years [13].
